# Descriptive analysis of durvalumab use in unresectable stage III non-small cell lung cancer in patients treated in Quebec’s University teaching hospitals

**DOI:** 10.3389/fonc.2024.1506172

**Published:** 2024-12-12

**Authors:** Valérie Labrie, Judith Lefebvre, Catherine Labbé, Kevin Jao, Mandy Malick, Serge Simard, Nicole Bouchard

**Affiliations:** ^1^ Centre Intégré Universitaire de Santé et de Services Sociaux de l’Estrie – Centre Hospitalier Universitaire de Sherbrooke (CIUSSS de l’Estrie-CHUS), Sherbrooke, QC, Canada; ^2^ Respirology Department, Institut Universitaire de Cardiologie et de Pneumologie de Québec (IUCPQ), Québec, QC, Canada; ^3^ Hôpital du Sacré-Cœur-de-Montréal, - Centre Intégré Universitaire de Santé et de Services Sociaux Nord de l’île de Montréal, Université de Montréal, Montréal, QC, Canada

**Keywords:** chemoradiotherapy, durvalumab, immunotherapy, non-small cell lung cancer, real-world data

## Abstract

**Introduction:**

Consolidation durvalumab post chemo-radiotherapy (CRT) has been demonstrated to improve survival in locally advanced non-small-cell lung cancer (NSCLC). Real-world data to assess its use and impact on patients, particularly in Quebec, remain limited.

**Methods:**

We, therefore, aimed to assess real-world durvalumab use in inoperable stage III NSCLC in Quebec, to describe progression-free survival (PFS) and overall survival (OS) outcomes as reported in the PACIFIC trial, and to evaluate safety and toxicity. Patients were retrospectively reviewed between January 1rst 2019 and December 31st 2020, based on their oncology reference date.

**Results:**

One hundred and eight patients treated with CRT were included, among which 82 received durvalumab (75.9%). The mean duration of treatment was 48 weeks [4-52], and 55% of patients completed the full treatment. Median PFS was 40 months in patients treated with CRT + durvalumab vs 6.9 months with CRT alone, with a hazard ratio (HR) of 0.22 (95% confidence interval (CI) 0.13-0.37; p < 0.0001). Limited access to CT scanning during the COVID pandemic, might have led to delayed disease progression detection and thus prolonged PFS. Median OS was > 52.8 months with CRT + durvalumab vs 19 months with CRT alone (HR 0.33, 95% CI 0.18-0.60; p=0.0002).

**Discussion:**

These findings support the efficacy and safety profile of durvalumab in real-world settings.

## 1 Introduction

According to the Canadian Cancer Society (1), lung cancer is the most diagnosed cancer, comprising 13% of new cancer cases each year and is also responsible for 24% of cancer-related mortality (1, 2). Twenty to thirty-five percent of non-small-cell lung cancer (NSCLC) are stage III at diagnosis (3).

In 2017, the phase III PACIFIC trial (4) changed the standard of care for unresectable NSCLC by adding durvalumab after concurrent CRT (5, 6).

Durvalumab is a monoclonal antibody that blocks the interaction between programmed cell death ligand 1 (PD-L1) and the programmed cell death protein 1 (PD-1) and CD-80 receptors. This increases the T-cells response against tumor cells (4).

The trial enrolled patients older than 18 years old who received two or more cycles of platinum-based chemotherapy and radiotherapy, and who had no disease progression after treatment, with an Eastern Cooperative Oncology Group (ECOG) performance status of 0 to 1. Durvalumab was initiated within 42 days after completion of CRT (4). Median PFS was 16.8 months (95% CI 13.0-18.1; p < 0.001) and the twelve-month PFS rate was 55.9% (4). Durvalumab was discontinued in a total of 15.4% of patients due to adverse events (4). Median OS was 47.5 months (4).

Since then, systematic reviews and meta-analyses have been published to assess the real-world use, safety, and toxicity of durvalumab (7–10). These studies indicated results consistent with the short-term effectiveness and safety of durvalumab as mentioned in the PACIFIC trial (8, 9). However, there are still few studies, often with limited patient numbers and lack of local data in the Quebec population (11–17).

Durvalumab has been used in Quebec, Canada since September 2018, initially through industry driven special access programs and subsequently via public reimbursement in February 2019 (7, 18). Data provided by the manufacturer concerning durvalumab in Quebec in 2021 revealed that among patients who completed CRT, 30% did not complete the twelve months of durvalumab consolidation (19, 20). The average duration of consolidation was 8.2 months (19). The reasons why durvalumab was not initiated or discontinued are unknown in the Quebec population.

Therefore, the aim of our study is to assess the real-world use of durvalumab in Quebec, Canada among patients with inoperable stage III NSCLC, to determine real-world PFS and OS, to identify factors influencing outcomes, and to evaluate safety and toxicity of this drug.

## 2 Materials and methods

### 2.1 Study design and patients

We conducted a retrospective observational multicenter cohort study involving one hundred and eight patients with inoperable stage III NSCLC from three Quebec University teaching hospitals. Patients were referred to oncology between January 1^st^, 2019, and December 31^st^, 2020. They were categorized based on whether they had received durvalumab or not. During the date range, all patients had access to durvalumab, initially through a special access program. The participating centers included Centre intégré de santé et des services sociaux de l’Estrie-Centre Hospitalier Universitaire de Sherbrooke (CIUSSS de l’Estrie CHUS), Institut Universitaire de Cardiologie et de Pneumologie de Québec (IUCPQ) and Hôpital du Sacré-Cœur-de-Montréal.

**Figure 1:**
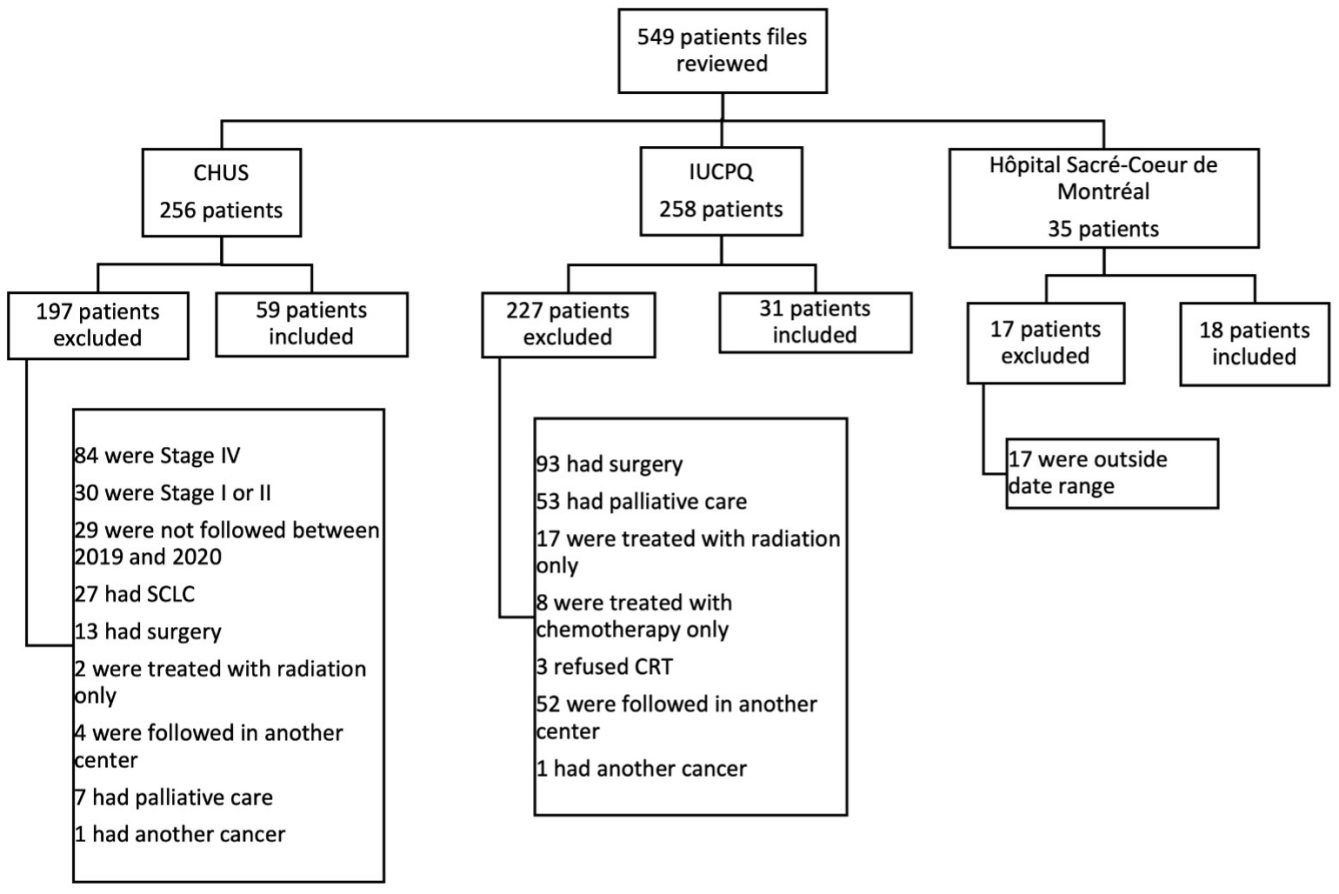
Flow chart. CHUS, Centre hospitalier universitaire de Sherbrooke; IUCPQ, Insitut Universitaire de cardiologie et de pneumologie de Québec; SCLC, small-cell lung cancer.

We included adult patients, ≥ 18 years, with unresectable stage III NSCLC at diagnosis in one of our three centers who completed curative intent CRT. Patients who continued their immunotherapy treatment in a community hospital where safety data were unavailable were excluded (**Figure 1**).

Recist evaluation was done per investigator. Patients had CT scans post completion of CRT.

### 2.2 Data collection

Data collection methods varied among centers. At CIUSSS de l’Estrie CHUS, a local research platform named Onco-expert was utilized. Patients records from archives were accessed to identified patients at IUCPQ and Hôpital du Sacré-Coeur-de-Montréal. Following approval from the Direction des Services Professionnels (DSP), we established a comprehensive database. This database was constructed using a systematic probabilistic sampling methodology, encompassing all patients who fulfilled our predetermined inclusion criteria. Patient data were meticulously extracted from their electronical medical records.

Data were obtained retrospectively and collected until August 2023. These included demographics, smoking status, cancer stage and histology, mutational status, PD-L1 expression, ECOG performance status before and after CRT, chemotherapy regimens, delay before initiation of durvalumab, duration of durvalumab treatment, adverse events, and reasons for not initiating or completing durvalumab treatment. Follow-up data were also collected, including the date of cancer progression, site of first recurrence and date of death (if applicable), or date of last follow-up. Cancer stage was determined according to the eighth edition TNM stage classification for lung cancer (21).

### 2.3 Ethical consideration

The project (MEO-2023-4898) received approval from the research ethics committees from IUCPQ, CIUSSS de l’Estrie CHUS and Hôpital du Sacré-Cœur-de-Montréal. A delegated consent process was defined as exempt from requiring individual patient consent.

### 2.4 Study measures

Our primary outcome was to assess real-world use of durvalumab in Quebec population, defined by the proportion of patients starting durvalumab after completion of CRT. Secondary outcomes included to determine the proportion of patients initiating durvalumab within 42 days of completion of CRT as described in the PACIFIC trial, to evaluate PFS, defined as the time from CRT start to the date of cancer progression, and OS defined as the time from CRT start to the date of death. Our definitions of PFS and OS differ from those used in the PACIFIC trial. In this trial, PFS was defined as the duration from randomization, occurring up to six weeks after CRT, to the date of the first documented event of tumor progression or death in the absence of disease progression (4). Overall survival was defined as the duration from randomization until death from any cause (4). To describe PFS, the date of cancer progression is used, or if no progression has occurred, the death date is considered. Censoring occurs at the last follow-up if the patient is alive and has not experienced cancer progression at the end of the study period. To describe OS, censoring occurs at last follow-up if the patient is alive at the end of the study period. For both analyses, the “cut-off” is defined as the date of death.

We also assessed cycle frequency (every 2 or 4 weeks), mean treatment duration and, if applicable, reasons for not initiating or completing durvalumab. Adverse events caused by immunotherapy and their grades were also part of our secondary outcomes. Toxicity grades were determined according to the National Cancer Institute Common Terminology Criteria for Adverse Events version 5.0 (22).

### 2.5 Statistical analysis

Patients were divided into two groups according to whether they received durvalumab consolidation or not. Descriptive analyses were used for the study population. The continuous variable “age” was expressed as mean ± standard deviation (SD). Categorical variables were presented as absolute and relative frequencies and were analyzed using the Fisher’s exact test. The OS and PFS analyses used the Nelson-Aalen estimator for the survival curves. Cox univariate regression models were used to explore candidate variables for the multivariable model building. The martingale residuals were used to examine the functional form of the continuous variable “age” and to conclude that no transformation was necessary. The graphical representation of the logarithm cumulative hazard rates versus time was used to assess the proportionality assumption of nominal variables. Second, an artificially time-dependent covariate was added to the univariate models to test the proportionality assumption. The proportional hazards assumption was not rejected as local tests linked to the time-dependent covariates were not significant and scatter plots were roughly constant over time for all variables. Variables from univariate analyses with a probability value <0.20 were candidates for the multivariate Cox regression model buildings for OS and PFS. The selection of variables was performed using a forward approach. The Akaike’s information criterion (AIC) and Schwarz’s Bayesian criterion (SBC) were used to compare candidate models. The final model includes variables associated with a p value < 0.05. Except when specified, the threshold for statistical significance was p< 0.05 for all analyses. Analyses were performed using SAS version 9.4 (SAS Institute Inc., Cary, NC).

## 3 Results

### 3.1 Characteristics of patients

We identified 108 patients who underwent CRT following diagnosis of unresectable stage III NSCLC (59 from CIUSSS de l’Estrie CHUS, 31 from IUCPQ and 18 from Hôpital du Sacré-Cœur-de-Montréal). Among them, 82 patients subsequently received durvalumab. Reasons for not initiating durvalumab in the CRT alone group were progression during or after CRT (n=15), comorbidities or poor ECOG PS (n=4), sequential treatment (n=2), patient preference (n=2), toxicity from CRT (n=1), and presence of an EGFR mutation (n=1).

Patients characteristics are summarized in **Table 1**. Of note, one patient in each group had an epidermal growth factor receptor (EGFR) mutation, one patient (1%) in the CRT + durvalumab group had an anaplastic lymphoma kinase (*ALK*) rearrangement and two patients (2%) also in the CRT + durvalumab group had a ROS1 mutation.

**Table 1:** Patient characteristics (n = 108).

	CRT + Durvalumab n = 82	CRT alone n = 26	P value
**Median age** **years** (mean ± SD)	65.5 ± 7.8	67.9 ± 7.7	0.16
**Male sex**	37 (45%)	14 (54%)	0.50
**Former or current smoker**	78 (95%)	26 (100%)	0.57
Race			
White Asian Other	80 (98%) 1 (1%) 1 (1%)	24 (92%) 0 2 (8%)	0.24
Disease stage (TNM 8^th^ edition)			
IIIA IIIB IIIC Not reported	40 (49%) 31 (38%) 6 (7%) 5 (6%)	10 (38%) 11 (42%) 3 (12%) 2(8%)	0.69
Histologic subtype			
Adenocarcinoma Squamous carcinoma NSCLC NOS	62 (76%) 19 (23%) 1 (1%)	14 (54%) 11 (42%) 1 (4%)	0.10
PD-L1 expression			
<1% 1-49% ≥ 50% Unknown	18 (22%) 23 (28%) 39 (48%) 2 (2%)	11 (42%) 4 (16%) 11 (42%) 0	0.21
*EGFR* mutation			
Positive Negative Not tested (including squamous carcinoma)	1 (1%) 70 (86%) 11 (13%)	1 (4%) 20 (77%) 5 (19%)	
Platinum agent			
Cisplatin Carboplatin	38 (46%) 44 (54%)	7 (27%) 19 (73%)	0.11
Second chemotherapy agent			
Pemetrexed^1^ Etoposide Paclitaxel Vinorelbine Gemcitabine	34 (41%) 26 (32%) 19 (23%) 3 (4%) 0	7 (27%) 4 (16%) 11 (42%) 2 (8%) 2 (8%)	0.01
**Concurrent CRT**	79 (96%)	16 (62%)	<0.0001
Radiation dose			
< 50 Gy 50-54 Gy 55 Gy ≥ 60 Gy	1 (1%) 4 (5%) 7 (9%) 70 (85%)	2 (8%) 6 (22%) 2 (8%) 16 (62%)	0.09
ECOG PS score at diagnosis			
0 1 2	36 (44%) 41 (50%) 5 (6%)	7 (27%) 15 (58%) 4 (15%)	0.15
ECOG PS after CRT			
0 1 2 3	11 (13%) 64 (79%) 6 (7%) 1 (1%)	6 (23%) 8 (31%) 9 (35%) 3 (11%)	<0.0001

CRT, chemoradiotherapy; ECOG PS, Eastern Cooperative Oncology Group performance status; EGFR, epidermal growth factor receptor; NSCLC NOS, non-small cell lung cancer not otherwise specified; PD-L1, programmed death-ligand 1; SD, standard deviation. 1: Non squamous only.

The only statistically significant differences between the two groups were the choice of the second chemotherapy agent, the use of concurrent vs sequential CRT, and the ECOG PS after CRT.

Median follow-up (IQR) in the durvalumab group was 3.0 years (1.9, 3.5) versus 1.1 years (0.5, 2.2) in the CRT alone group.

### 3.2 Characteristics of durvalumab treatment

Median time from CRT completion to the initiation of durvalumab was 38 days [15-116] (**Table 2**). Fifty-four patients (66%) started durvalumab within the 42-day window, consistent with the PACIFIC trial (4). Median treatment duration was 48 weeks [4-52]. Among the 82 patients receiving durvalumab, 55% (n = 45) completed the full course of durvalumab treatment, while 15% (n = 12) discontinued due to disease progression, 25% (n = 21) stopped treatment due to adverse events, and 5% (n =4) stopped for other reasons. Cycle frequency varied significantly, with 30 patients receiving durvalumab every 4 weeks, 28 patients undergoing treatment every 2 weeks and 24 patients transitioning from biweekly to monthly dosing schedules. Some of those changes were due to COVID-19 pandemic, trying to minimize hospital visits for patients.

**Table 2:** Characteristics of durvalumab treatment.

	N = 82
Median time from CRT completion to durvalumab start, days [range]	38 [15-116]
Time from CRT completion to durvalumab start ≤ 42 days	54 (66%)
Weight-based dosing	57 (70%)
Cycle frequency	
Every 4 weeks Every 2 weeks Switched from every 2 to every 4 weeks	30 (37%) 28 (34%) 24(29%)
Median treatment duration, weeks [range]	48 [4-52]
Durvalumab treatment	
Completed Stopped for progression Stopped for adverse events Stopped for other reasons	45 (55%) 12 (15%) 21 (25%) 4 (5%)

### 3.3 Efficacy

PFS and OS were based on follow-up data. The first dose of Durvalumab was administered on February 5, 2019 and the last on May 16, 2021. The most recent follow-up in our database was on August 28^th^, 2023 marking the end of the data collection. For patients who died, the follow up duration ranged from 2.5 months to 46.2 months (3.9 years). For the majority of patients still alive, regardless on whether they received durvalumab, the follow up was at least 2.15 years.

#### 3.3.1 Progression-free survival

**Figure 2:**
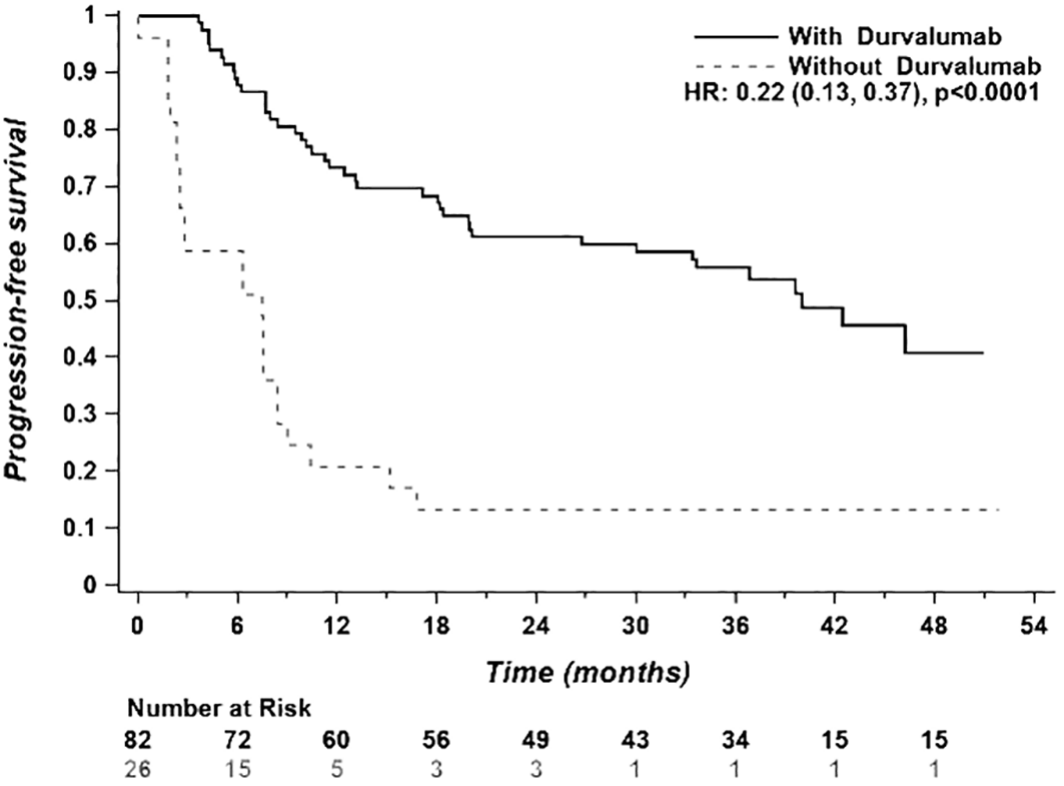
Progression-free survival of the entire study population (n = 108).

**Figure 3:**
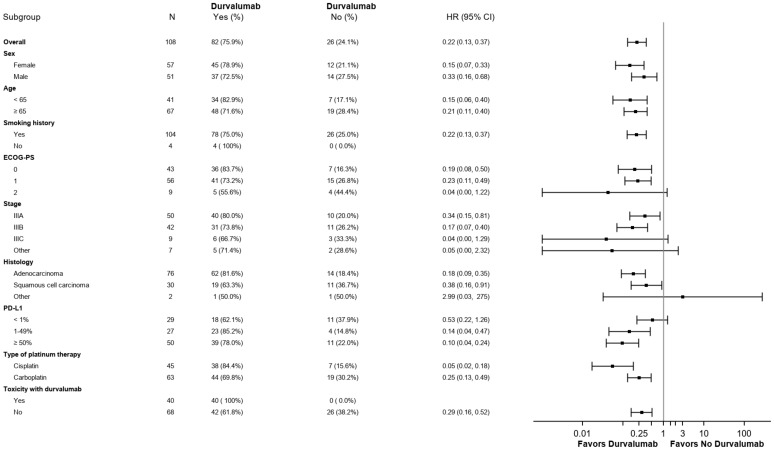
Subgroup analysis of prognostic factors for progression-free survival.

Median PFS in the CRT + durvalumab group was 40.0 months (95% CI 20.1->51) vs 6.9 months (95% CI 2.5-8.4) in the CRT alone group (HR 0.22; 95% CI: 0.13-0.37, p< 0.0001) (**Figure 2**). Multivariate analyses using Cox regression were conducted to identify factors influencing PFS (**Figure 3**). Sex and age (< 65 years vs ≥65 years) did not impact PFS significantly. ECOG performance status of 0 and 1 (HR 0.19, 95% CI: 0.08-0.50 and HR 0.23, 95% CI: 0.11-0.49, respectively) favored the CRT + durvalumab group. Patients with PD-L1 expression of 1-49% (HR 0.14, 95% CI: 0.04-0.47) and ≥ 50% (HR 0.10, 95% CI: 0.04; 0.24) experienced significantly increased PFS. All stages and histology subtypes benefited from durvalumab treatment except the other histology group which included poorly differentiated NSCLC and large cell lung carcinoma. Regarding chemotherapy regimens, both platinum-based chemotherapies showed benefit with durvalumab, particularly those containing cisplatin. In **Figure 2**, for patients without durvalumab, only 3 patients out of 26 have been censored, and these censored observations are at 25.8, 27.5 and 51.9 months, corresponding to the three longest follow-ups; for patients with treatment, 41 patients (50%) have been censored and the first patient to be censored was at 22.8 months.

#### 3.3.2 Overall survival

**Figure 4:**
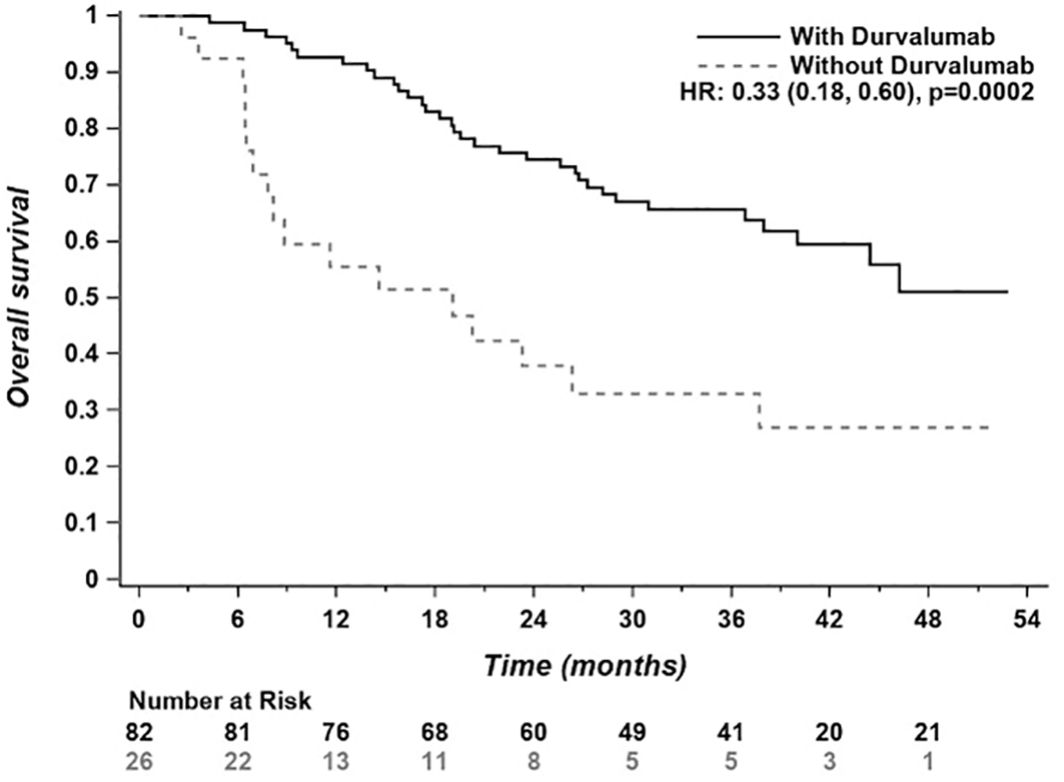
Overall survival of the entire study population (n = 108). Progression/Subtx; Progression with subsequent treatment; Progression/No Sub tx, Progression with no subsequent treatment.

**Figure 5:**
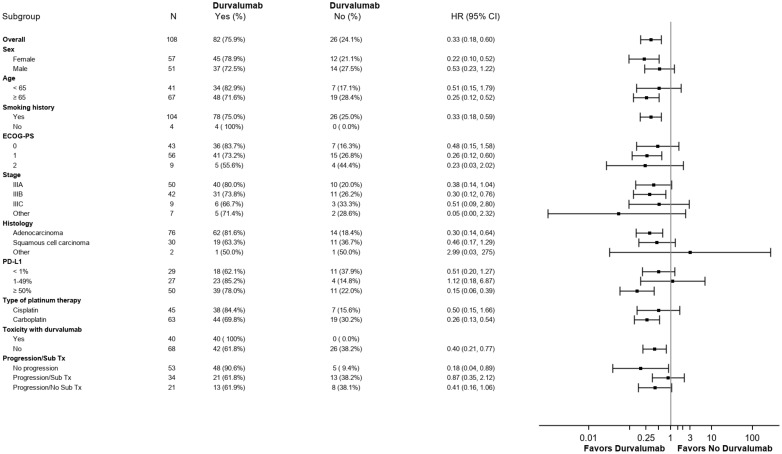
Subgroup analysis of prognosis factors for overall survival.

Median OS in the CRT + durvalumab group was > 52.8 months vs 19.0 months (95% CI 6.9-37.7) in the CRT alone group (HR 0.33; 95% CI: 0.18-0.60, p=0.0002). (**Figure 4**). Unlike PFS, multivariate analyses (**Figure 5**) revealed an improvement in overall survival with female gender (HR 0.22, 95% CI: 0.10-0.52) and age ≥ 65 years (HR 0.25, 95% CI: 0.12-0.52). PD-L1 expression ≥ 50% favored OS with durvalumab use (HR 0.15, 95% CI: 0.06-0.39). In contrast, to the PACIFIC study, PD-L1 expression of 1-49% was not statistically significant regarding overall survival.

### 3.4 Adverse events

Immune-related adverse events (IRAEs) were reported in 40/82 patients who initiated durvalumab. Among these subjects, 29 had only one adverse event; 9 had two adverse events and 2 had three adverse events. Regarding adverse events, 31.7% were grade 1, 36.6% were grade 2, 26.8% were grade 3 and 4.9% were grade 4. The most frequently observed IRAE was pulmonary toxicity (n = 18). Durvalumab treatment was discontinued in 25% of patients (n = 21) due to toxicity. Detailed toxicities are presented in **Table 3**. Other toxicities included infusion reaction (Grades 2 and 3) and meningoencephalitis (Grade 4).

**Table 3:** Adverse events.

Toxicity	Grade 1 (n = 13)	Grade 2 (n = 15)	Grade 3 (n = 11)	Grade 4 (n = 2)	Total (n = 41)
Rash	4	0	0	0	4
Hypothyroidism	3	3	1	0	7
Colitis	2	1	2	0	5
Hepatitis	1	0	1	0	2
Pneumonitis	2	9	6	1	18
Arthritis	0	1	0	0	1
Nephritis	1	0	0	0	1
Other toxicities	0	1	1	1	3

## 4 Discussion

Our study provides insights into the real-world utilization of durvalumab in patients with unresectable stage III NSCLC treated across three Quebec university teaching hospitals. Our objectives included describing PFS and OS and assessing the safety and toxicity of durvalumab following curative CRT, compared to the findings from PACIFIC trial (4).

Our study population is representative of the Quebec population. It was conducted with patients from the three largest pulmonary oncology centers in Quebec, located in three cities with university hospital centers.

The median age of patients receiving durvalumab in our study (65.5 years) closely aligns with the median age reported in the PACIFIC trial (64 years) (4). Most patients in our CRT + durvalumab group (96%) received concurrent CRT, in contrast to 62% in the CRT alone group. In comparison, the PACIFIC trial reported 99% of patients receiving concurrent CRT in both study arms, consistent with the study protocol. Patients who do not undergo concurrent CRT are typically less medically fit and therefore may not be candidates for either concurrent CRT or additional immunotherapy. It might have been anticipated that the median age in our real-world study would have been higher, considering typical demographics. Moreover, most patients in our study received concurrent CRT, in contrast to other real-world settings where sequential CRT is also common alongside durvalumab use. Our patients were treated before publications showing comparable safety profile and encouraging preliminary efficacy with durvalumab after sequential chemoradiation (23).

Median PFS in the CRT + durvalumab group (40.0 months) notably exceeded the findings from the PACIFIC trial (16.9 months) and from PACIFIC-R study (24) where the median real-worlds PFS was 21.7 months (23.7 months with concurrent CRT vs 19.3 months with sequential CRT), reaffirming durvalumab’s efficacy in a real-world setting. This disparity may be attributed, in part, to factors such as limited access to computed tomography (CT) scanning during the pandemic, potentially leading to delayed disease progression detection and thus prolonged PFS. The standard practice in Quebec university teaching hospitals is to conduct chest CT scans every three months. There was variability between the three centers regarding the intervals at which follow-up CT scans were performed and we don’t have the exact information per center. We can only assume that the CT scans might have been performed at longer intervals because of the pandemic. On March 11, 2020 the World Health Organization (WHO) declared COVID-19 viral disease a pandemic. On May 4, 2023, the International Health Regulations (IHR) Emergency Committee of the WHO downgraded the COVID-19 pandemic, as the first patient received durvalumab on February 5, 2019 and the last on May 16, 2021.

This frequency contrasts with the PACIFIC trial (4), where follow-up CT scans were scheduled every eight weeks. This variation could also explain our observed longer PFS compared to the PACIFIC trial results. Moreover, we calculated the PFS from the beginning of chemoradiotherapy, which is earlier than the PACIFIC study. Our findings regarding PFS are consistent with those reported in a real-world study conducted by Mooradian et al. (25) indicating a HR 0.36 (95% CI 0.26-0.51). Our study also observed a more pronounced benefit in patients with PD-L1 ≥ 50%. Also, the CRT alone group might also have included patients who were not candidates for durvalumab because of poor ECOG PS or poor prognosis. This could have led to an overestimation of the PFS and also OS when compared to the durvalumab group.

In terms of median OS, it exceeded 52.8 months with CRT + durvalumab compared to 19.0 months with CRT alone (HR 0.33, 95% CI 0.18-0.60; p=0.0002). Contrasting this, the PACIFIC trial (4) reported a HR for median OS of 0.68 (99.73% CI 0.47-0.997). In the five years updated analysis by Spiegel et al. (10) the stratified HR for OS was 0.72 (95% CI 0.59-0.89) with a median OS of 47.5 months vs 29.1 months. This variance could potentially be attributed to our inclusion criteria, which encompassed all patients who received CRT, including those who experienced progression shortly after and consequently did not qualify for durvalumab, as opposed to the PACIFIC trial (4), which exclusively enrolled non progressing patients. Our findings regarding OS are consistent with those reported in a real-world study conducted by Mooradian et al. (25) indicating a HR 0.27 (95% CI 0.16-0.43). The OS may also be affected by subsequent treatments, such as access to therapies including immune checkpoint inhibitors (IO) or tyrosine kinase inhibitors (TKI). It remains uncertain whether all patients included in the PACIFIC study had access to the full spectrum of potential treatments.

Regarding durvalumab prescription, only 66% of patients (n = 54) initiated durvalumab within 42 days of completing chemoradiation therapy, with a median delay of 38 days. Reasons for these delays were multifactorial, including CT scan delays, poor patient performance status, and impacts from the COVID-19 pandemic (27). Cycle frequency varied, predominantly between 2- and 4-week intervals, influenced by pandemic-related healthcare adaptations, risk of adverse events and patient preferences. Interestingly, 55% of our patients completed the 1-year duration of durvalumab treatment, which is higher than reported in the PACIFIC-R study (47.1%) (24).

Primary reasons for durvalumab cessation included disease progression (15%) and adverse events (25%), notably pneumonitis. A substantial proportion of patients experiencing grade 2 to 4 pneumonitis (n = 11/18) did not resume treatment. Our rate of discontinuation was higher than in the PACIFIC-R study, which was only 16.5% (24).

Our safety profile is similar to the PACIFIC trial, with adverse events occurring in 48.8% of patients receiving durvalumab (4). Pneumonitis was our most common adverse event, affecting 26.8% of patients (n= 18), with grade 3 or higher pneumonitis observed in 8.5%, higher than reported in the original study. In PACIFIC trial (4), grade 3 or 4 pneumonitis occurred only in 3.4% and 2.6%. Higher rates of pneumonitis leading to treatment discontinuation had already been published for real world evidence in the PACIFIC-R Study (24) (any-grade: 18.5%) and in a systematic review and meta-analysis (8–10). Comparable rates of other toxicities such as skin, endocrine, and gastrointestinal toxicities were noted. No deaths due to adverse events were reported in our retrospective study.

Spigel et al. (10) provides further evidence of durvalumab’s efficacy and safety in inoperable stage III NSCLC, highlighting its enduring benefits five years after the PACIFIC trial (10). Also, Denault et al. (26) conducted a retrospective cohort study on OS and PFS with durvalumab use in the British Columbian population. It was demonstrated that durvalumab improved OS in the PD-L1 ≥ 1% group (HR 0.53 95% CI 0.34-0.81 p = 0.003). This improvement was not demonstrated when PD-L1 was less than 1%. Their findings align with our study, as our patient cohort primarily consists of individuals who underwent concurrent rather than sequential CRT, demonstrating improved outcomes notably in cases with higher PD-L1 expression levels.

Our study has some limitations. Our retrospective study had a recall bias among patients from Hôpital du Sacré-Cœur-de-Montréal, impacting inclusion rates. Those patients were identified by medical oncologists who were treating them and were included in our retrospective study. At other centers, such as IUCPQ and CIUSSS de l’Estrie CHUS, all patients diagnosed with non-operable stage III NSCLC who underwent CRT were included. Additionally, the study small sample size may have impacted statistical power. Duration of patient follow-up is short for the evaluation of median survival. Radiological CT scan follow-up was disrupted during the pandemic, probably affecting PFS measurement. Adverse events were identified by chart review, and less severe side effects were possibly less reported. However, oncology pharmacy notes were also consulted to identify adverse events that may have been missed by the medical team.

## 5 Conclusions

In conclusion, our retrospective study confirms the effectiveness and safety profile of durvalumab following curative CRT for stage III unresectable NSCLC within an entirely public health system. The results also demonstrate improved outcomes with higher PD-L1 expression levels. Despite the challenges posed by COVID pandemic, the majority of patients were able to maintain their treatment regimen and their follow-up protocols.

## Data Availability

The original contributions presented in the study are included in the article/supplementary material. Further inquiries can be directed to the corresponding author.
